# Personal attributes that influence the adequate management of hypertension and dyslipidemia in patients with type 2 diabetes. Results from the DIAB-CORE Cooperation

**DOI:** 10.1186/1475-2840-11-120

**Published:** 2012-10-05

**Authors:** Ina-Maria Rückert, Werner Maier, Andreas Mielck, Sabine Schipf, Henry Völzke, Alexander Kluttig, Karin-Halina Greiser, Klaus Berger, Grit Müller, Ute Ellert, Hannelore Neuhauser, Wolfgang Rathmann, Teresa Tamayo, Susanne Moebus, Silke Andrich, Christa Meisinger

**Affiliations:** 1 Institute of Epidemiology II, Helmholtz Zentrum München, German Research Center for Environmental Health (GmbH) and German Center for Diabetes Research (DZD e.V.), Ingolstädter Landstrasse 1, D-85764, München/Neuherberg, Germany; 2 Institute of Health Economics and Health Care Management, Helmholtz Zentrum München, German Research Center for Environmental Health (GmbH), Neuherberg, Germany; 3 Institute for Community Medicine, University Medicine Greifswald, Greifswald, Germany; 4 Institute of Medical Epidemiology, Biostatistics and Informatics, Martin-Luther-University Halle-Wittenberg, Halle, Saale, Germany; 5 German Cancer Research Centre, Division of Cancer Epidemiology, Heidelberg, Germany; 6 Institute of Epidemiology and Social Medicine, University of Muenster, Muenster, Germany; 7 Department of Epidemiology and Health Reporting, Robert-Koch-Institute, Berlin, Germany; 8 Institute of Biometrics and Epidemiology, German Diabetes Center, Leibniz Center for Diabetes, Research at Heinrich-Heine-University Düsseldorf, Düsseldorf, Germany; 9 Institute of Medical Informatics, Biometry and Epidemiology, University Hospital of Essen, University of Duisburg-Essen, Essen, Germany; 10 MONICA/KORA Myocardial Infarction Registry, Central Hospital of Augsburg, Augsburg, Germany

**Keywords:** Type 2 Diabetes, Comorbidities, Hypertension, Dyslipidemia, Adherence to guidelines, Sex differences

## Abstract

**Background:**

Hypertension and dyslipidemia are often insufficiently controlled in persons with type 2 diabetes (T2D) in Germany. In the current study we evaluated individual characteristics that are assumed to influence the adequate treatment and control of hypertension and dyslipidemia and aimed to identify the patient group with the most urgent need for improved health care.

**Methods:**

The analysis was based on the DIAB-CORE project in which cross-sectional data from five regional population-based studies and one nationwide German study, conducted between 1997 and 2006, were pooled. We compared the frequencies of socio-economic and lifestyle factors along with comorbidities in hypertensive participants with or without the blood pressure target of < 140/90 mmHg. Similar studies were also performed in participants with dyslipidemia with and without the target of total cholesterol/HDL cholesterol ratio < 5. Furthermore, we compared participants who received antihypertensive/lipid lowering treatment with those who were untreated. Univariable and multivariable logistic regression models were used to assess the odds of potentially influential factors.

**Results:**

We included 1287 participants with T2D of whom n = 1048 had hypertension and n = 636 had dyslipidemia. Uncontrolled blood pressure was associated with male sex, low body mass index (BMI), no history of myocardial infarction (MI) and study site. Uncontrolled blood lipid levels were associated with male sex, no history of MI and study site. The odds of receiving no pharmacotherapy for hypertension were significantly greater in men, younger participants, those with BMI < 30 kg/m2 and those without previous MI or stroke. Participants with dyslipidemia received lipid lowering medication less frequently if they were male and had not previously had an MI. The more recent studies HNR and CARLA had the greatest numbers of well controlled and treated participants.

**Conclusion:**

In the DIAB-CORE study, the patient group with the greatest odds of uncontrolled co-morbidities and no pharmacotherapy was more likely comprised of younger men with low BMI and no history of cardiovascular disease.

## Introduction

### Background

Hypertension and dyslipidemia constitute major public health problems as they increase the risk of cardiovascular diseases (CVDs), especially in patients with concomitant type 2 diabetes (T2D) [1-4].

Nevertheless, several epidemiological studies indicate disappointing deficiencies in the detection and adequate treatment of hypertension and dyslipidemia in Germany with minimal improvement over the last decades [5-13]. About ten years ago, the South German site of the Monitoring Trends and Determinants in Cardiovascular Disease (MONICA) Study, the forerunner to the Cooperative Health Research in the Region of Augsburg (KORA) Study, revealed that the proportion of hypertensive participants with controlled hypertension < 140/90 mmHg was only 7% in men and 13% in women [6]. An earlier analysis of regional differences using data of the two population-based German studies KORA and SHIP (Study of Health in Pomerania) with participants aged 25-74 years found that of all hypertensive participants, 8.4% in SHIP and 10.2% in KORA, were treated and well controlled. Recently, data of the German DIAB-CORE cooperation revealed that 64% of all participants with T2D and 49% of participants without T2D, aged 45-74 years, had untreated or insufficiently treated hypertension [8]. Despite the increased awareness of physicians and the public regarding the beneficial effects of blood pressure control on cardiovascular outcomes such as myocardial infarction and stroke ( [5]), the data suggest that further barriers must exist that hinder optimal health care delivery.

### Objectives

To our knowledge, no population-based study in Germany has so far dealt with characteristics of patients with T2D and hypertension and/or dyslipidemia who are not sufficiently treated for these serious conditions. In our study, we describe demographic, socio-economic and lifestyle factors in DIAB-CORE participants with diabetes and concomitant hypertension or dyslipidemia and analyse the factors associated with insufficient disease control and absent pharmacotherapy. We therefore aim to identify the group of patients with the most urgent need for intensified care.

## Methods

### Study design and setting

The DIAB-CORE Consortium consists of the following population-based studies (from north to south): the Study of Health in Pomerania (SHIP, Greifswald), the Dortmund Health Study (DHS, Dortmund), the Cardiovascular Disease, Living and Ageing (CARLA, Halle (Saale)) Study, the Heinz Nixdorf-Recall (HNR, Risk Factors, Evaluation of Coronary Calcification, and Lifestyle, Bochum, Essen, Mülheim a. d. Ruhr) Study, the Cooperative Health Research in the Region of Augsburg (KORA, Augsburg) Study, and the nationwide German National Health Interview and Examination Survey 1998 (GNHIES98), see Table 1.

**Table 1:** Studies included in the pooled DIAB-CORE sample (45–74 years)

**Study**	**Region**	**Study period**	**N (%)**	**Age (years)**	**Hypertension**	**Dyslipidemia**
				**mean (SD)**	**N (%)**	**N (%)**
**SHIP**^**a**^	North-east	1997–2001	251 (19.5)	62.8 (7.5)	215 (85.7)	143 (57.0)
	Germany					
	(West Pomerania)					
**DHS**^**b**^	West Germany	2003–2004	87 (6.8)	64.1 (8.0)	73 (83.9)	-
	(Dortmund)					
**CARLA**^**c**^	East Germany	2002–2006	174 (13.5)	63.5 (7.3)	155 (89.6)	84 (48.6)
	(Halle)					
**HNR**^**d**^	West Germany (Bochum, Essen, Mülheim an der Ruhr)	2000–2003	350 (27.2)	63.0 (7.2)	263 (78.5)	174 (52.1)
**KORA**^**e**^	South Germany	1999–2001	146 (11.3)	63.3 (6.7)	114 (78.6)	80 (55.9)
	(Augsburg region)					
**GNHIES98**^**f**^	Nationwide	1997–1999	279 (21.7)	62.7 (6.8)	228 (81.7)	155 (59.6)
**Total**		**1997–2006**	**1287**	**63.1 (7.2)**	**1048 (82.5)**	**636 (54.8)**

^a^SHIP: Study of Health in Pomerania; ^b^DHS: Dortmund Health Study; ^c^CARLA: Cardiovascular Disease, Living and Ageing in Halle; ^d^ HNR: Heinz Nixdorf-Recall; ^e^KORA (Survey S4): Cooperative Health Research in the Region of Augsburg; ^f^GNHIES98: German National Health Interview and Examination Survey 1998.

BP > = 140/90 mmHg or using anti-hypertensive medication.

TC/HDL > = 5 or using lipid-lowering medication.

All studies were conducted between 1997 and 2006 and used similar instruments, questionnaires and medical measurements to assess data. Detailed descriptions of study designs, samples and procedures are available elsewhere [14-20]. Ethical approval was obtained for each study. Primary study data of interest were pooled and frequencies compared.

### Variables

#### ***Age***

Participants were classified in five age groups using five year intervals.

#### ***Type 2 diabetes***

T2D was defined based on self-report or self-reported intake of oral anti-diabetic agents, insulin or a combination of both. Some studies lacked information on diabetes type. Thus, in order to exclude participants who probably had Type 1 diabetes, self-reported age at diagnosis of diabetes was used, and only those patients with an age at diagnosis of > 30 years were included in the T2D group.

#### ***Hypertension***

Hypertension was defined using the mean of the second and third blood pressure measurements (the first and second measurements in DHS) conducted at the study centres with systolic blood pressure ≥140 and/or diastolic blood pressure ≥90 mmHg, or intake of anti-hypertensive medication in participants with physician’s diagnosis of hypertension (“awareness”). Participants with hypertension were categorized into one of the following four subgroups: (1) aware (with physician’ diagnosis) and controlled treated to target levels of < 140/90 mmHg, (2) aware and treated, but not reaching target blood pressure values of < 140/90 mmHg, i.e. uncontrolled treated, (3) aware, but not treated, (4) unaware of hypertension. Thus, “awareness” of hypertension applied to participants in categories 1, 2 and 3, “treatment” applied to those in categories 1 and 2 and “control” to those in category 1.

#### ***Dyslipidemia***

Total cholesterol, high-density lipoprotein (HDL) cholesterol and low-density lipoprotein (LDL) cholesterol levels were measured from random blood samples. Dyslipidemia was defined analogously to hypertension using information on lipid-lowering medication intake, self-reported information on physician's diagnosis and a total cholesterol to HDL cholesterol ratio (TC/HDL) of > = 5 [21,22].

#### ***Myocardial infarction (MI) and stroke***

Self-reported data on myocardial infarction and stroke (“Did you ever have a myocardial infarction/stroke, diagnosed by a physician?”) was assessed identically in all studies.

#### ***Anti-hypertensive medication***

All study participants were asked to bring original packaging of their medications used during the last seven days to the examination. The variable “anti-hypertensive medication” included any prescription of medication belonging to the ATC subgroups C02 (antihypertensives), C03 (diuretics), C04 (peripheral vasodilators), C07 (beta blocking agents), C08 (calcium channel blockers) and C09 (agents reacting on the renin-angiotensin system).

#### ***Lipid-lowering medication***

Medications of the ATC subgroup C10 (lipid modifying agents) were included in the variable “lipid-lowering medication”.

#### ***Body mass index (BMI)***

The BMI (kg/m2) was calculated using standardized weight and height measurements.

#### ***Smoking***

Two categories (current vs. ex- and never smoker) were defined to differentiate risk types. A current smoker reported smoking at least one cigarette per day. Persons who had reported that they had smoked at least one cigarette per day in the past but who quit smoking at least one year ago were defined as ex-smokers. Never-smokers were defined as those persons who had never smoked or smoked only occasionally (< 1 cigarette day).

#### ***Physical inactivity***

In all studies, physical activity was assessed by self-report only. A threshold of less than 1 h of physical activity per week was determined for a high risk lifestyle. Assessment of activity was restricted to all kinds of exercise training but did not comprise low level exercise such as stepping stairs or walking, as this type of exercise was not assessed in all studies.

#### ***Educational level***

In all studies, the participants were asked for their highest level of school qualification obtained. We classified educational level as a dichotomous variable contrasting low with medium or high level. According to the German school system, low educational level includes participants with up to 9 years of schooling. Medium educational level is equivalent to 10 years of schooling and high educational level to 12 or 13 years of schooling, which is required to enter a university.

#### ***Income***

Information on monthly net household income as well as on household size was obtained from interviews. As the ages of household members were not available consistently across all studies, it was not possible to calculate the equivalent income according to the OECD equivalence scale. That is why the equivalent income was calculated according to the Luxembourg Income Study (income/household size) [23]. Pooling of income data was conducted by a regional approach, calculating the median income for each of the study centers separately. This approach allowed us to take into account overall income differences between the regions. For each study, we differentiated three income groups (<60% of the study-specific median income, ≥60% up to 150%, >150%) and pooled these groups across the six studies.

### Participants

The pooled data set included 1,287 participants with T2D aged 45 to 74 years. In the nationwide survey GNHIES98, 3% non-German citizens were included, KORA, SHIP and CARLA focused on participants of German nationality, and the other studies collected information on birthplace only. Two-stage cluster sampling or stratified random sampling were used. Overall response ranged between 56 and 69%.

The hypertension sub-analysis included 1,048 hypertensive participants. N = 17 participants were excluded due to missing information on: study blood pressure measurement (n = 3), physician’s diagnosis of hypertension (n = 3), and/or medication intake (n = 14).

The dyslipidemia sub-analysis included 636 participants with dyslipidemia according to the definition specified above. N = 126 participants had to be excluded due to missing information on laboratory measurements (n = 32), physician’s diagnosis of dyslipidemia (n = 90, all participants of DHS and 3 of HNR), and/or medication intake (n = 14). N = 16 participants were not included in either one of the two sub-analyses.

### Statistical analyses

T2D participants with hypertension, with or without the blood pressure target of < 140/90 mmHg, and T2D participants with dyslipidemia with or without the target of total cholesterol/HDL cholesterol < 5, as well as T2D participants with or without treatment were compared with respect to their lifestyle factors, cardiovascular burden, clinical measurements and medications. Continuous variables were characterized by means and standard deviations (SD), categorical variables were described as percentages. Differences between groups were calculated using t-tests and Wilcoxon tests (continuous variables) or chi-square tests and univariable logistic regression models (categorical variables). At first, univariable logistic regression models were programmed to identify factors associated with blood pressure, lipid ratio or medication intake. In a second step, variables found to be significantly associated with the respective outcome were included in multivariable models to examine adjusted effects.

The goodness-of-fit of adjusted models was assessed by the c-value, and the Hosmer-Lemeshow test. The c-value describes the area under the ROC-curve, ranging from 0.5 (random correlations) to 1.0 (perfect fit). In epidemiologic studies, values between 0.6 and 0.8 are usually regarded as satisfactory. The Hosmer-Lemeshow test compares observed and predicted values with each other. If there is no significant difference (at the significance level of 0.05) the model is characterized as being appropriate.

A two-sided alpha level of 0.05 was chosen as criterion for statistical significance. All analyses were carried out using SAS, version 9.2 (SAS Institute Inc., Cary, NC, USA).

## Results

### Participants

Of 1,287 T2D participants aged 45-74 years, n = 1,048 (82.5%) had hypertension and n = 636 (54.8%) had dyslipidemia. N = 530 (41.2%) had both conditions and were included in both sub-analyses.

The frequency of hypertension in persons with T2D differed slightly between studies, ranging from 78.5% in HNR to 89.6% in CARLA. Likewise, the number of participants with dyslipidemia ranged from 48.6% in CARLA to 59.6% in GNHIES98. The mean age was 63.1 years (Std. 7.2 years), and was very similar across all studies (Table 1). In total, 45.1% of participants were female, ranging from 40.0% in HNR to 49.8% in GNHIES98. Within the group of hypertensive participants, 46.3% were female, among participants with dyslipidemia, 43.1% were female.

### Descriptive data

**Figure 1:**
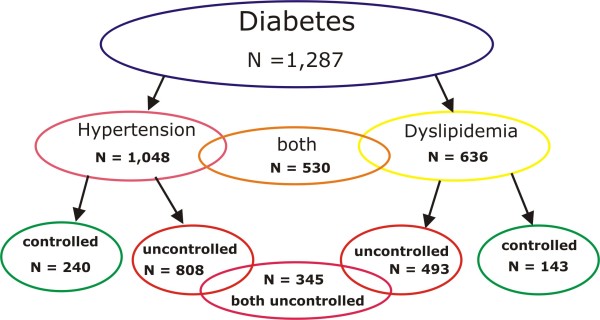
Frequencies of controlled and uncontrolled hypertension and dyslipidemia.

Of 1,048 participants with T2D and hypertension, n = 240 (22.9%) had controlled blood pressure of < 140/90 mmHg, and n = 808 (77.1%) were uncontrolled. Dyslipidemia was treated to TC/HDL ratio < 5 in 143 (22.5%) of 636 T2D participants and uncontrolled dyslipidemia was observed in 493 (77.5%) T2D participants (Figure 1).

Frequencies of associated variables, stratified by participants with and without controlled hypertension and with and without controlled dyslipidemia, respectively, are shown in Table 2. Frequencies of participant characteristics, stratified by those treated for hypertension or dyslipidemia, respectively, and those not treated for these diseases are shown in Table 3.

**Table 2:** Study characteristics of participants with T2D in DIAB-CORE, age range 45-74

	**Controlled hypertension < 140/90 mmHg**	**Uncontrolled hypertension > = 140/90 mmHg**	**Controlled dyslipidemia TC/HDL ratio < 5**	**Uncontrolled dyslipidemia TC/HDL ratio > =5**
	**n = 240**	**n = 808**	**n = 143**	**n = 493**
Age (years)	62.8 (7.2)	63.8 (6.9)	63.8 (6.7)	62.4 (7.3)
Women (%)	59.6	42.3	54.6	39.8
BMI (kg/m2)	32.4 (5.9)	30.7 (5.0)	30.6 (5.1)	31.2 (5.2)
BMI > = 30 kg/m2 (%)	64.2	48.6	51.1	50.7
Current smoking (%)	15.7	14.2	13.4	19.9
Low physical activity (%)	71.2	73.5	68.1	75.0
Low income (%)	17.8	17.0	17.4	18.5
Low education (%)	78.3	78.7	80.9	79.6
Diabetes duration (years)	8.3 (7.7)	8.9 (7.5)	10.1 (7.9)	7.9 (7.0)
**Diabetes treatment (%)**				
Diet only or no treatment (%)	20.0	21.9	20.3	21.5
OAD only (%)	47.5	51.2	42.7	53.7
Insulin only (%)	19.6	17.1	19.6	13.4
OAD and Insulin (%)	12.9	9.8	17.5	11.4
**Blood pressure (mm Hg)**				
Systolic BP (mm Hg)	126.3 (10.4)	160.0 (16.4)	148.1 (23.9)	147.6 (21.6)
Diastolic BP (mm Hg)	75.7 (7.9)	87.8 (10.7)	80.6 (11.1)	84.1 (11.4)
**Hypertension (%)**	100.0	100.0	85.9	82.8
**current BP > =140/90 (%)**	0.0	100.0	59.9	65.9
**Cholesterol (mg/dl)**				
TC (mg/dl)	218.4 (46.2)	226.8 (50.3)	199.0 (37.0)	244.5 (53.6)
LDL (mg/dl)	132.3 (37.9)	138.5 (39.9)	114.4 (32.4)	153.9 (42.1)
HDL (mg/dl)	50.2 (15.7)	50.0 (16.0)	56.3 (15.5)	39.1 (8.4)
**Dyslipidemia (%)**	53.8	55.9	100.0	100.0
**current TC/HDL ratio > = 5 (%)**	37.1	43.0	0.0	100.0
Myocardial infarction (%)	13.4	9.4	22.7	11.7
Stroke (%)	9.2	7.4	7.0	7.7

Numbers are means (SD) or percentages and relate to the number of subjects available for analysis.

*BMI*: body mass index, *OAD*: oral anti-diabetic medication, *TC*: total cholesterol, *LDL*: low-density lipoprotein, *HDL*: high-density lipoprotein, *CVD*: cardiovascular disease, *BP*: blood pressure.

BP > =140/90 mmHg or using anti-hypertensive medication.

TC/HDL ratio > =5 or using lipid-lowering medication.

Test of the difference between participants with controlled hypertension and those with uncontrolled hypertension, p <0.05.

Test of the difference between participants with controlled dyslipidemia and those with uncontrolled dyslipidemia, p <0.05.

**Table 3:** Characteristics of diabetic participants with and without medical treatment for hypertension or dyslipidemia

	**Anti-hypertensive medication**	**No anti-hypertensive medication**	**Lipid lowering medication**	**No lipid lowering medication**
	**n = 822**	**n = 226**	**n = 240**	**n = 396**
Age (years)	64.1 (6.8)	61.8 (7.3)	63.5 (6.5)	62.2 (7.5)
Women (%)	50.1	32.3	51.7	37.9
BMI (kg/m2)	31.5 (5.4)	29.5 (4.2)	30.8 (5.1)	31.2 (5.2)
BMI > = 30 kg/m2 (%)	56.0	38.2	51.3	50.5
Current smoking (%)	12.8	20.9	14.8	20.6
Low physical activity (%)	73.4	71.4	69.1	76.0
Low income (%)	16.8	18.4	18.3	18.2
Low education (%)	79.4	75.7	81.3	79.0
Diabetes duration (years)	9.0 (7.6)	7.8 (7.0)	9.2 (7.3)	7.9 (7.2)
**Diabetes treatment (%)**				
Diet only or no treatment (%)	19.6	28.3	18.3	23.0
OAD only (%)	49.5	53.5	46.3	54.2
Insulin only (%)	19.1	12.4	19.6	11.9
OAD and Insulin (%)	11.8	5.8	15.8	10.9
**Blood pressure (mm Hg)**				
Systolic BP (mm Hg)	150.4 (21.6)	159.0 (16.0)	147.3 (23.3)	148.0 (21.4)
Diastolic BP (mm Hg)	83.7 (11.3)	90.0 (9.7)	80.9 (11.2)	84.8 (11.4)
**Hypertension (%)**	100	100	87.0	81.3
**current BP > =140/90 (%)**	70.8	100 (due to definition of hypertension)	60.7	66.9
**Cholesterol (mg/dl)**				
TC (mg/dl)	224.9 (51.2)	224.6 (43.0)	215.6 (50.2)	245.5 (52.8)
LDL (mg/dl)	136.8 (40.1)	138.0 (37.2)	126.9 (42.3)	155.3 (40.5)
HDL (mg/dl)	49.9 (15.8)	50.3 (16.3)	48.8 (15.8)	39.4 (8.5)
**Dyslipidemia (%)**	56.1	52.9	100	100
**current TC/HDL ratio > = 5 (%)**	40.9	44.6	40.4	100 (due to definition of dyslipidemia)
Myocardial infarction (%)	12.1	3.6	23.1	8.7
Stroke (%)	9.1	3.1	8.0	7.3

Numbers are means (SD) or percentages and relate to the number of subjects available for analysis.

*BMI*: body mass index, *OAD*: oral anti-diabetic medication, *TC*: total cholesterol, *LDL*: low-density lipoprotein, *HDL*: high-density lipoprotein, *CVD*: cardiovascular disease, *BP*: blood pressure.

BP > =140/90 mmHg or using anti-hypertensive medication.

TC/HDL ratio > =5 or using lipid-lowering medication.

Test of the difference between participants with antihypertensive medication and those without such medication, p <0.05.

Test of the difference between participants with lipid lowering medication and those without such medication, p <0.05.

### Focus on differences between women and men

**Figure 2:**
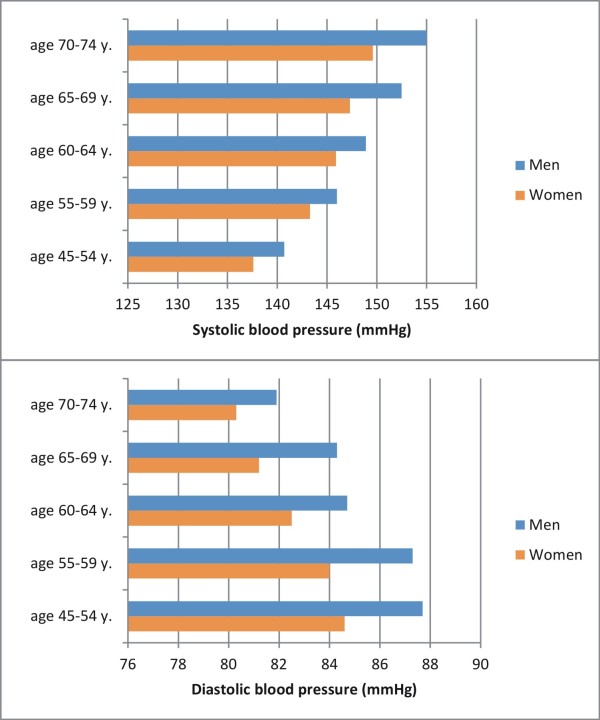
Systolic and diastolic blood pressure in all women and men with T2D stratified by age groups.

**Figure 3:**
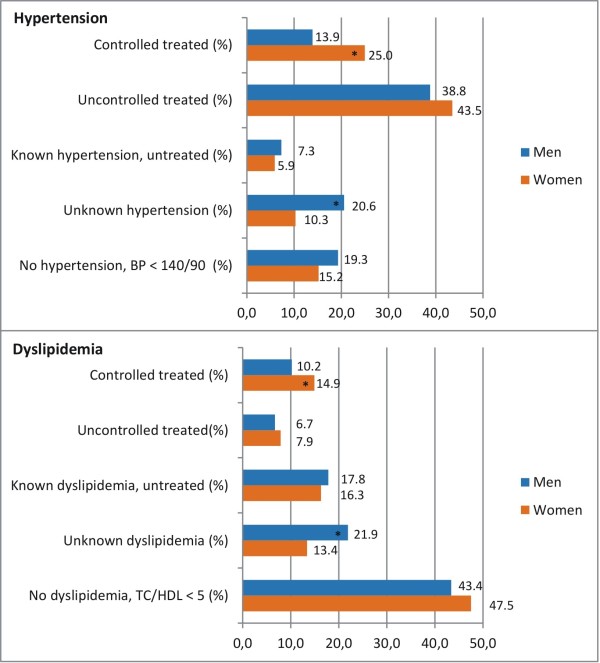
Percentages of all participants with T2D - without hypertension or without dyslipidemia, with controlled, uncontrolled treated, known, but not treated, and unrecognized disease status stratified by sex.

Systolic blood pressure (SBP) increased with age in participants with T2D, while diastolic blood pressure (DBP) decreased. Both measurements were consistently lower in women of all age groups than in their male counterparts (mean SBP in women: 149.8 mmHg (SD 20.8) vs. 154.4 mmHg (20.6) in men, mean DBP 83.2 (11.1) and 86.6 (11.2) in women and men, respectively) (Figure 2). Whilst men were more often free from hypertension (19.3% vs. 15.2%), women were significantly more often diagnosed and well controlled if affected: 25.0% of all women had controlled hypertension, compared to 13.9% in men, 43.5% were treated but did not achieve goal levels (compared to 38.8% in men), 5.9% were not treated even though they had a physician’s diagnosis of hypertension (compared to 7.3% in men), and 10.3% had unknown hypertension (compared to 20.6% in men) (see Figure 3).

Total cholesterol and HDL-cholesterol were higher in women than in men (232.2 mg/dl (SD 48.8) vs. 216.4 mg/dl (48.0) and 53.9 (16.6) vs. 46.3 (14.0)) – albeit, 48.7% of women and 35.1% of men had less than 50 or less than 40 mg/dl HDL respectively. However, the TC/HDL ratio was lower in women (4.7 (1.7) vs. 5.0 (1.7)).

More women than men were not affected with dyslipidemia as defined by a TC/HDL ratio < 5 (47.5% vs. 43.4%), 14.9% were treated and well-controlled (vs. 10.2% in men), 7.9% were treated but did not reach goal values (compared to 6.7% in men), 16.3% were not treated despite of a physician’s diagnosis (compared to 17.8% in men) and 13.4% had unknown dyslipidemia (compared to 21.9% in men) (see Figure 3).

### Factors associated with uncontrolled hypertension or uncontrolled dyslipidemia

Univariable logistic regression models yielded significant associations of uncontrolled hypertension with sex (OR for men: 2.01, 95% CI 1.50-2.69), BMI < 30 (OR = 1.89, 95% CI 1.40-2.55), and study. Age in five groups was not significantly associated but displayed a steady tendency towards having the greatest odds in the second oldest group (65-69 years) compared to the youngest group (45-54 years). With GNHIES98 as the reference study, HNR had the smallest odds of uncontrolled hypertension (OR = 0.32, 95% CI 0.20-0.50), followed by CARLA (OR = 0.47, 95% CI 0.28-0.79) and KORA (OR = 0.52, 95% CI 0.30-0.91). Multivariable analysis with these significant variables (i.e. sex, BMI and study) showed consistent results as well as the associated factor ‘no previous MI’ which reached statistical significance (OR = 1.70, 95% CI 1.06-2.70). A model including only sex, BMI and study had a c-value of 0.68 and a non-significant Hosmer-Lemeshow test (p = 0.78).

Uncontrolled dyslipidemia was significantly associated with sex (OR for men: 1.82, 95% CI 1.25-2.65), no previous MI (OR = 2.22, 95% CI 1.37-3.59) and study (OR for CARLA: 0.24, 95% CI 0.13-0.46 and HNR 0.41, 95% CI 0.23-0.74, GNHIES98 as reference). Again, age was not significantly associated, but showed some tendency, albeit in the other direction, with the youngest age group displaying the greatest odds of uncontrolled dyslipidemia (70-74 years as reference). Multivariable analysis with sex, MI, and study generated consistent odds. A model including only these variables had a c-value of 0.69 and a non-significant Hosmer-Lemeshow test result (p = 0.96). Smoking, physical activity, educational level and income were not significantly associated with either control of hypertension or dyslipidemia (Table 4).

**Table 4:** Factors associated with high BP* and cholesterol^ in diabetic study participants with concomitant (diagnosed or unrecognized) hypertension or dyslipidemia

	**Hypertension**				**Dyslipidemia**			
	**N**	**BP > =140/90**	**Unadjusted**	**Adjusted for Sex, BMI, Study**	**N**	**TC/HDL ratio > = 5**	**Unadjusted**	**Adjusted for Sex, MI, Study**
		**n (%)**				**n (%)**		
	**1048**	**808 (77.1)**	**OR (95%CI)**	**OR (95%CI)**	**636**	**493 (77.5)**	**OR (95%CI)**	**OR (95%CI)**
**Age in years**								
70-74	257	202 (78.6)	1.45 (0.90-2.35)	1.48 (0.90-2.46)	134	101 (75.4)	Reference	Reference
65-69	252	201 (79.8)	1.56 (0.96-2.54)	**1.70 (1.02-2.84)**	146	105 (71.9)	0.84 (0.49-1.43)	0.83 (0.47-1.45)
60-64	254	196 (77.2)	1.34 (0.83-2.15)	1.33 (0.81-2.19)	157	126 (80.3)	1.33 (0.76-2.32)	1.28 (0.71-2.30)
55-59	151	113 (74.8)	1.18 (0.70-1.99)	1.13 (0.65-1.96)	98	79 (80.6)	1.36 (0.72-2.57)	1.23 (0.63-2.40)
45-54	134	96 (71.6)	Reference	Reference	101	82 (81.2)	1.41 (0.75-2.66)	1.33 (0.70-2.59)
**Sex**								
Male	563	466 (82.8)	**2.01 (1.50-2.69)**	**2.00 (1.47-2.72)**	362	297 (82.0)	**1.82 (1.25-2.65)**	**2.23 (1.49-3.33)**
Female	485	342 (70.5)	Reference	Reference	274	196 (71.5)	Reference	Reference
**BMI**								
> = 30	546	392 (71.8)	Reference	Reference	322	249 (77.3)	Reference	Reference
<30	500	414 (82.8)	**1.89 (1.40-2.55)**	**1.75 (1.28-2.40)**	312	242 (77.6)	1.01 (0.70-1.47)	0.82 (0.55-1.23)
**Currently smoking**								
Yes	150	113 (75.3)	Reference	Reference	115	96 (83.5)	1.61 (0.94-2.74)	1.56 (0.88-2.75)
No	880	682 (77.5)	1.13 (0.75-1.69)	1.30 (0.84-1.99)	510	387 (75.9)	Reference	Reference
**Physical inactivity**								
Yes	757	589 (77.8)	1.13 (0.82-1.55)	0.98 (0.70-1.38)	461	365 (79.2)	1.40 (0.93-2.11)	1.27 (0.82-1.98)
No	280	212 (75.7)	Reference	Reference	167	122 (73.1)	Reference	Reference
**School education**								
Low	811	627 (77.3)	1.02 (0.72-1.46)	1.07 (0.73-1.55)	499	385 (77.2)	Reference	Reference
High and middle	221	170 (76.9)	Reference	Reference	126	99 (78.6)	1.09 (0.68-1.74)	1.00 (0.60-1.64)
**Income**								
Low	162	124 (76.5)	Reference	Reference	104	81 (77.9)	Reference	Reference
Middle	674	521 (77.3)	1.04 (0.70-1.57)	0.86 (0.56-1.33)	405	306 (75.6)	0.88 (0.52-1.47)	0.84 (0.49-1.45)
High	109	86 (78.9)	1.15 (0.64-2.06)	0.98 (0.53-1.83)	62	52 (83.9)	1.48 (0.65-3.35)	1.12 (0.47-2.65)
**Myocardial infarction**								
Yes	107	75 (70.1)	Reference	Reference	89	57 (64.0)	Reference	Reference
No	934	727 (77.8)	1.50 (0.96-2.33)	**1.70 (1.06-2.70)**	540	431 (79.8)	**2.22 (1.37-3.59)**	**2.81 (1.69-4.69)**
**Stroke**								
Yes	81	59 (72.8)	Reference	Reference	48	38 (79.2)	1.11 (0.54-2.28)	1.19 (0.56-2.55)
No	960	742 (77.3)	1.27 (0.76-2.12)	1.63 (0.95-2.79)	585	453 (77.4)	Reference	Reference
**Dyslipidemia**					**Hypertension**			
Yes	530	410 (77.4)	1.09 (0.81-1.47)	1.01 (0.73-1.38)	530	408 (77.0)	Reference	Reference
No	426	323 (75.8)	Reference	Reference	105	85 (81.0)	1.27 (0.75-2.15)	1.32 (0.75-2.31)
**Study**								
GNHIES98	228	195 (85.5)	Reference	Reference	155	135 (87.1)	Reference	Reference
CARLA	155	114 (73.6)	**0.47 (0.28-0.79)**	**0.49 (0.29-0.83)**	84	52 (61.9)	**0.24 (0.13-0.46)**	**0.21 (0.11-0.40)**
DHS	73	63 (86.3)	1.07 (0.50-2.29)	1.10 (0.51-2.39)	-	-	-	**-**
KORA	114	86 (75.4)	**0.52 (0.30-0.91)**	0.57 (0.32-1.02)	80	63 (78.8)	0.55 (0.27-1.12)	0.52 (0.25-1.07)
HNR	263	172 (65.4)	**0.32 (0.20-0.50)**	**0.30 (0.19-0.47)**	174	128 (73.6)	**0.41 (0.23-0.74)**	**0.38 (0.21-0.69)**
SHIP	215	178 (82.8)	0.81 (0.49-1.36)	0.84 (0.50-1.41)	143	115 (80.4)	0.61 (0.33-1.14)	0.63 (0.33-1.20)

Uncontrolled hypertension – Sex, BMI, Study: ROC = 0.68, Hosmer-Lemeshow p-value = 0.78.

Uncontrolled dyslipidemia – Sex, MI, Study: ROC = 0.69, Hosmer-Lemeshow p-value = 0.96.

*BP > = 140/90 mm Hg.

^ ratio total cholesterol/HDL cholesterol > = 5.

### Factors associated with no pharmacotherapy for hypertension or dyslipidemia

Using univariable regression models, we found significant odds of non-use of anti-hypertensive medication in younger study participants (OR for 45-54 years: 2.54, 95% CI 1.54-4.20, OR for 55-59 years: 2.15, 95% CI 1.31-3.53, 70-74 years as reference), males (OR = 2.11, 95% CI 1.54-2.87), current smokers (OR: 1.80, 95% CI 1.22-2.64) and participants with BMI < 30 (OR = 2.06, 95% CI 1.52-2.79). Moreover, no previous MI (OR = 3.75, 95% CI 1.79-7.82), no previous stroke (OR = 3.12, 95% CI 1.42-6.88) and study (OR for CARLA: 0.42, 95% CI 0.24-0.71, OR for HNR: 0.60, 95% CI 0.40-0.92, GNHIES98 as reference) were significantly associated with untreated hypertension. In multivariable models adjusted for age, sex, BMI, MI, stroke, and study, the odds remained significant, except for smoking. A model including only these significant variables was characterized by a c-value of 0.71 and a non-significant Hosmer-Lemeshow test result (p = 0.46).

Non-use of lipid lowering medication in participants with dyslipidemia was univariably associated with male sex (OR = 1.75, 95% CI 1.27-2.43) and no MI (OR = 3.16, 95% CI 1.99-5.01). Multivariable analysis adjusted for these two variables yielded additionally significant odds for no concomitant hypertension (OR = 1.71, 95% CI 1.06-2.78), physical inactivity (OR = 1.49, 95% CI 1.03-2.17) and the CARLA study (OR = 0.56, 95% CI 0.32-0.98). A model including only sex and MI had a c-value of 0.64 and a non-significant Hosmer-Lemeshow test (p = 0.62). Neither education nor income were associated with treatment of hypertension or dyslipidemia (Table 5).

**Table 5:** Factors associated with absent pharmacotherapy in diabetic study participants with concomitant (diagnosed or unrecognized) hypertension or dyslipidemia

		**Hypertension**				**Dyslipidemia**		
	**N**	**Without anti-hypertensive medication n (%)**	**Unadjusted**	**Adjusted for Age, Sex, BMI, MI, Stroke, Study**	**N**	**Without lipid lowering medication n (%)**	**Unadjusted**	**Adjusted for Sex, MI**
	**1048**	**226 (21.6)**	**OR (95%CI)**	**OR (95%CI)**	**636**	**396 (62.3)**	**OR (95%CI)**	**OR (95%CI)**
**Age in years**								
70-74	257	38 (14.8)	Reference	Reference	134	84 (62.7)	Reference	Reference
65-69	252	49 (19.4)	1.39 (0.87-2.21)	1.35 (0.83-2.19)	146	81 (55.5)	0.74 (0.46-1.20)	0.71 (0.43-1.16)
60-64	254	57 (22.4)	**1.67 (1.06-2.62)**	1.46 (0.90-2.35)	157	97 (61.8)	0.96 (0.60-1.55)	0.94 (0.57-1.54)
55-59	151	41 (27.2)	**2.15 (1.31-3.53)**	**1.80 (1.06-3.05)**	98	63 (64.3)	1.07 (0.62-1.84)	0.93 (0.53-1.63)
45-54	134	41 (30.6)	**2.54 (1.54-4.20)**	**2.28 (1.34-3.90)**	101	71 (70.3)	1.41 (0.81-2.45)	1.10 (0.62-1.95)
**Sex**								
Male	563	153 (27.2)	**2.11 (1.54-2.87)**	**2.11 (1.52-2.93)**	362	246 (68.0)	**1.75 (1.27-2.43)**	**2.02 (1.44-2.84)**
Female	485	73 (15.1)	Reference	Reference	274	150 (54.7)	Reference	Reference
**Smoking**								
Yes	150	46 (30.7)	**1.80 (1.22-2.64)**	1.40 (0.92-2.13)	115	80 (69.6)	1.49 (0.96-2.30)	1.30 (0.82-2.04)
No	880	174 (19.8)	Reference	Reference	510	309 (60.6)	Reference	Reference
**BMI**								
> = 30	546	86 (15.8)	Reference	Reference	322	199 (61.8)	Reference	Reference
<30	500	139 (27.8)	**2.06 (1.52-2.79)**	**1.98 (1.43-2.74)**	312	195 (62.5)	1.03 (0.75-1.42)	0.89 (0.63-1.24)
**Physical inactivity**								
Yes	757	160 (21.1)	Reference	Reference	461	298 (64.6)	1.42 (0.99-2.04)	**1.49 (1.03-2.17)**
No	280	64 (22.9)	1.11 (0.80-1.54)	1.10 (0.77-1.56)	167	94 (56.3)	Reference	Reference
**School education**								
Low	811	168 (20.7)	Reference	Reference	499	308 (61.7)	Reference	Reference
High and middle	221	54 (24.4)	1.24 (0.87-1.76)	1.08 (0.74-1.58)	126	82 (65.1)	1.16 (0.77-1.74)	0.98 (0.64-1.50)
**Income**								
Low	162	37 (22.8)	Reference	Reference	104	65 (62.5)	Reference	Reference
Middle	674	133 (19.7)	0.83 (0.55-1.26)	0.73 (0.47-1.14)	405	248 (61.2)	0.95 (0.61-1.48)	0.92 (0.58-1.47)
High	109	31 (28.4)	1.34 (0.77-2.34)	0.90 (0.49-1.65)	62	45 (72.6)	1.59 (0.80-3.15)	1.17 (0.57-2.39)
**Myocardial infarction**								
Yes	107	8 (7.5)	Reference	Reference	89	34 (38.2)	Reference	Reference
No	934	217 (23.2)	**3.75 (1.79-7.82)**	**3.65 (1.72 -7.75)**	540	357 (66.1)	**3.16 (1.99-5.01)**	**3.62 (2.25-5.82)**
**Stroke**								
Yes	81	7 (8.6)	Reference	Reference	48	29 (60.4)	Reference	Reference
No	960	219 (22.8)	**3.12 (1.42-6.88)**	**3.45 (1.53-7.79)**	585	366 (62.6)	1.10 (0.60-2.00)	0.99 (0.52-1.85)
**Dyslipidemia**					**Hypertension**			
Yes	530	109 (20.6)	Reference	Reference	530	322 (60.8)	Reference	Reference
No	426	97 (22.8)	1.14 (0.84-1.55)	1.15 (0.83-1.60)	105	74 (70.5)	1.54 (0.98-2.43)	**1.71 (1.06-2.78)**
**Study**								
GNHIES98	228	65 (28.5)	Reference	Reference	155	102 (65.8)	Reference	Reference
CARLA	155	22 (14.2)	**0.42 (0.24-0.71)**	**0.39 (0.22-0.69)**	84	46 (54.8)	0.63 (0.37-1.08)	**0.56 (0.32-0.98)**
DHS	73	17 (23.3)	0.76 (0.41-1.41)	0.91 (0.47-1.74)	-	-	**-**	-
KORA	114	24 (21.1)	0.67 (0.39-1.14)	0.82 (0.46-1.45)	80	52 (65.0)	0.97 (0.55-1.70)	0.95 (0.53-1.72)
HNR	263	51 (19.4)	**0.60 (0.40-0.92)**	**0.60 (0.39-0.93)**	174	101 (58.1)	0.72 (0.46-1.13)	0.68 (0.42-1.08)
SHIP	215	47 (21.9)	0.70 (0.46-1.08)	0.73 (0.46-1.16)	143	95 (66.4)	1.03 (0.64-1.66)	1.08 (0.65-1.79)

No pharmacotherapy for hypertension – Age, Sex, BMI, MI, Stroke, Study: ROC = 0.71, Hosmer-Lemeshow p-value = 0.46.

No pharmacotherapy for dyslipidemia – Sex, MI: ROC = 0.64, Hosmer-Lemeshow p-value = 0.62.

## Discussion

### Key results

Uncontrolled blood pressure in T2D participants with (diagnosed or unrecognized) hypertension was associated with male sex, BMI < 30 kg/m2, no previous MI, and study site. Similarly, uncontrolled blood lipid levels in T2D participants with dyslipidemia (diagnosed or unrecognized) were more frequent in men, those who had not suffered MI and attendees of the older DIAB-CORE studies.

Lack of treatment for hypertension was related to younger age, male sex, smoking, BMI < 30, no history of MI or stroke, and study site. Male T2D participants without concomitant hypertension and who had not suffered MI had significantly greater odds of untreated dyslipidemia than other participants in DIAB-CORE.

Socioeconomic features such as educational level and income were not significantly associated with either disease control or pharmacotherapy; lifestyle factors were only associated in some models.

On the one hand, the results of our analyses indicate that the vast majority of patients with T2D in Germany are not adequately treated for hypertension and/or dyslipidemia, even though these conditions have been found to considerably increase the risk of co-morbidities and complications such as MI, stroke, nephropathy and retinopathy.

On the other hand, we confirmed the presumption that patients with additional risk factors, such as advanced age, previous MI, previous stroke or obesity are treated more often for hypertension and dyslipidemia and (apart from those with older age) more frequently achieve adequate blood pressure and lipid target levels. The difference between female and male participants was pronounced and consistent over all sub-analyses.

### Strengths and limitations

The essential strength of our study is the large population-based sample drawn from the general German population, aged 45 to 74 years, and the fact that both, laboratory measurements and information on medication intake were available.

Differences in the frequencies of hypertension and dyslipidemia between studies were probably due to the relatively small numbers of affected participants within the individual studies and analyses. Due to the pooling process, only similarly collected and coded data of all six studies could be used and the least common denominator had to be found. Therefore, the definition of diabetes was based on self-report of physician’s diagnosis and treatment with anti-diabetic agents rather than on clinical diagnosis and medical records. Blood pressure was calculated using the mean of the second and third measurements in all studies except for DHS, where only two measurements were performed and used to calculate the mean.

Moreover, measurements of blood pressure and lipids based on a single testing opportunity provide evidence for the respective condition, but are not equal to a clinical diagnosis with repeated measurements. We cannot exclude cases of ‘white coat hypertension’, i.e. elevated blood pressure caused by the excitement of the unfamiliar situation.

Due to compliance issues, women might have reported their medication intake more reliably than men, thus pharmacotherapy in men could have been underestimated and consequently, sex-specific differences overestimated in these analyses.

### Generalization

Patient-centered studies in Germany and other countries have so far examined awareness, treatment, control and factors associated with insufficient control of co-morbidities in patients with and without diabetes. A systematic review by McLean et al. from 2008, which included 26 studies from different countries with 66,833 diabetes patients with co-morbid hypertension, concluded that 83% (range 32-100%) of patients were treated, yet only about 29% (range 5-59%) had their blood pressure controlled to < 140/90 mmHg. The proportions of treatment to control were similar and thus equally insufficient between studies and countries. Unfortunately, the authors did not report person-related factors associated with disease control [24].

### Differences by gender

There are very few studies that have reported factors associated with cardiovascular disease control in patients with diabetes, as opposed to adults in general, irrespective of diabetes status. However, a number of recent studies that focused on gender differences [25-30] found that female patients with diabetes had a worse cardiovascular risk profile and were less controlled compared to their male counterparts. Our study confirmed these results in part; women in DIAB-CORE had higher total cholesterol values than men. However, since HDL-cholesterol values were also higher, the TC/HDL ratio was lower and thus more beneficial in women. In all studies [25-30], blood pressure was higher in women, especially in patients with cardiovascular disease. Although this sex difference was abrogated in some analyses after adjustment for associated variables [27,28]. There was either no sex difference in the amount of antihypertensive and lipid-lowering medication, or women took more medication, which is in agreement with our study. Furthermore, our results indicate that men are about twice as often unaware of their hypertension and dyslipidemia as women, which is well in line with known gender differences concerning health behaviour. Especially in the age group of about 30 to 60 years, men use health care services less than women and tend to undervalue health care and health behaviour [31,32]. Several authors hypothesize that societally dominant ‘traditional masculinity’ leads men to adopt beliefs and behaviours that increase health risks and support the ideal of the ‘bulletproof superhero’ who would be embarrassed to check his cholesterol level [33-35].

### Influence of age

Findings on the influence of age on hypertension control are controversial (e.g. [36,37]). An explanation for less control with simultaneously intensified treatment, as seen in our study, might be that resistant hypertension occurs more often in older persons [38]. Accordingly, this association was not detected for dyslipidemia. A cross-sectional study in Sweden examined the assumption that the excess cardiovascular risk of persons with diabetes compared to persons without diabetes decreases with increasing age. However, the authors found that the burden of CVD risk factors clustered over the entire life span with increasing glucometabolic disturbance, especially in older women. Likewise, self-rated health decreased with increasing cardio-metabolic risk and age. The authors suggest that a decreased burden of risk in older patients with diabetes might be due to a survival bias [39].

### Association with body mass index

In our study obese participants (BMI ≥ 30 kg/m2) received medication more frequently and were more often well treated for hypertension, although they were more often affected with hypertension than leaner individuals (data not shown). A Swedish population-based study in hypertensive 60-year-old persons with and without diabetes by Carlsson et al. found an inverse, direct association of high waist circumference with uncontrolled hypertension [40]. The same seems to be true for patients in primary care irrespective of diabetes status [41]. Bramlage et al. found that the odds of good blood pressure control in diagnosed and treated individuals was significantly smaller in overweight and obese primary care patients than in patients with normal weight. Using data of physician diagnosed hypertensive DIAB-CORE participants without diabetes (n = 5012), we found that 29.2% of those with BMI < 25 had controlled hypertension (41.2% were uncontrolled and 29.6% untreated) compared to 28.6% of those with BMI ≥ 30 (50.9% uncontrolled and 20.5% untreated). These results indicate that obese people may be more difficult to treat and obtain goal blood pressure than lean persons; however, they appear to be more aware of their disease. The same seems to be true for a comparison of hypertensive people with and without T2D. Those with T2D are more often treated and well-controlled, much more often treated but not controlled, half as often untreated and half as often unrecognized, irrespective of BMI group (data not shown) than those without T2D. All in all, obese individuals do more often present with a blood pressure ≥ 140/90 mmHg, irrespective of treatment. Thus, people with additional risk factors (such as obesity and diabetes) may be more aware of their blood pressure and more often treated but at the same time, they are more difficult to effectively treat.

### Effect of socio-economic differences

We expected to find a significant negative association of high socio-economic status with uncontrolled co-morbidities, however no significant associations were observed with any socio-economic characteristics and the outcome variables. This might, in the case of school education, be due to the unequal frequencies of low (about 80%) vs. high and middle status, which owns to the high mean age of the study population (63 years) and the fact that older people mostly attended junior high school only. In the previously mentioned Swedish study, lack of health care due to low income was independently associated with uncontrolled hypertension in men (OR = 2.71, 95% CI 1.09-6.78) but not in women. In contrast, living in an apartment instead of a house (as an indicator of lower socio-economic status) remained a significantly protective factor in an adjusted model in women (OR = 0.55, 95%CI 0.35-0.85). The authors stated that the finding was puzzling and offered no explanation [40].

### Association with previous cardiovascular disease

Carlsson et al. confirmed that previous cardiovascular disease or coronary heart disease has a protective effect on uncontrolled hypertension, probably because of more intense treatment, better medication adherence and/or symptom relief of angina pectoris [40].

### Influence of more complex factors

There are a number of possible influences on uncontrolled hypertension and dyslipidemia that we could not consider. For example, the Swedish study by Carlsson et al. included data on nutrition and found that daily intake of fruit had an independent, protective effect in men but not in women [40].

Steckelings, 2004 et al. [5] used data from primary care patients, irrespective of diabetes status (the HYDRA Study: Hypertension and Diabetes Screening and Awareness Study), to describe possible determinants of unsatisfactory hypertension control in Germany. Less than 30% of treated and 19% of all patients, treated or untreated, had controlled blood pressure <140/90 mmHg. The frequency of diagnosis was particularly low in young people, probably due to insufficient blood pressure screening. The authors found that physicians used outdated guidelines and based treatment on diastolic pressure. The great majority of participants (94%) stated that they knew that hypertension is an important risk factor for serious diseases, and most of them (63%) occasionally measured their own blood pressure. However, the physicians participating in the study often misclassified their patients as ‘well controlled’ even though they had BP measurements ≥ 140/90 mmHg.

According to a review by Düsing et al. 2006 [42], more complex, hindering factors could also be insufficient education and motivation provided to the patient by physicians, patients’ reluctance to change lifestyle factors or commence/modify drug treatment, lack of awareness of the risks associated with hypertension, and poor compliance. The latter is a complex and thoroughly explored concept and challenge (e.g. [43]). Apparently, many people with hypertension do not seem to recognize high blood pressure as a progressive chronic illness, but rather misinterpret it as a risk factor in a gamble with a potentially positive outcome [44,45].

Finally, the sex of the attending physician has been shown to play a role in the quality of risk factor control in patients with hypertension and dyslipidemia. In a cross-sectional study by Journath et al., diabetic men and women achieved goals for blood pressure control, and men achieved goals for cholesterol control more often if they were treated by female physicians [46]. Likewise, a German study including 51,053 diabetes patients treated by 3,096 physicians concluded that female physicians achieved a better quality of care than their male counterparts, especially in risk management important for future disease prognosis [47].

### Differences between Europe and the United States/Canada

Interestingly, there appears to be an intriguing difference in hypertension prevalence and control, irrespective of diabetes status, between Europe and the United States/Canada that has not been appreciated and examined sufficiently. Wolf-Maier et al. [48,49] compared sample surveys conducted in the 1990s in Germany, Finland, Sweden, England, Spain, Italy, Canada, and the United States and found that even though mean BMI was very similar across these countries, the prevalence of hypertension, defined as blood pressure > 140/90 mmHg or intake of anti-hypertensive medication, differed remarkably. The European average was 44.2% compared with 27.6% in North America. Germany had the highest prevalence with 55.3% [48]. Treatment and control of hypertensive participants within the individual studies was also significantly better in the United States than in Europe with 7.8% hypertension control in the population (29.9% in treated hypertensive participants) in Germany compared to 28.6% (54.5% in treated hypertensives) in the United States [49].

In order to improve awareness, creative new approaches have been successfully implemented in the United States, e.g. by addressing the issue and counseling black men during a visit at a barbershop [50]. Such innovative ideas might also work in Germany – possibly for younger men with diabetes and no previous cardiovascular complications.

## Conclusions

In the DIAB-CORE study, participants with diabetes who were at the greatest odds of uncontrolled co-morbidities and no pharmacotherapy were male, younger, had lower BMI and no history of cardiovascular disease. Although the general risk profile appears relatively low, preventative efforts should not overlook, but specifically consider this group of patients.

## Abbreviations

ATC: Anatomical Therapeutic Chemical Classification System; BMI: Body mass index; BP: Blood pressure; CARLA: Cardiovascular Disease Living and Ageing Study; CVD: Cardiovascular disease; DHS: Dortmund Health Study; DMP: Disease management program; GNHIES98: German National Health Interview and Examination Survey; HDL: High-density lipoprotein; KORA: Cooperative Health Research in the Region of Augsburg Study; LDL: Low-density lipoprotein; OAD: Oral anti-diabetic medication; HNR: Heinz Nixdorf-Recall Risk Factors Evaluation of Coronary Calcification and Lifestyle Study; SD: Standard deviation; SHIP: Study of Health in Pomerania; T2D: Type 2 diabetes; TC: Total cholesterol.

## Competing interests

The authors declare that they have no competing interests.

## Authors' contributions

I-MR performed the statistical analyses and wrote the manuscript. CM and WM worked on pooling of the data, contributed to the discussion and reviewed/edited the manuscript. AM contributed to the discussion and reviewed/edited the manuscript. TT and WR worked on pooling of the data and reviewed/edited the manuscript. SS, HV, AK, K-HG, KB, GM, UE, HN, SM and SA contributed data and reviewed/edited the manuscript. All authors read and approved the final manuscript.
